# Quantification of thoracic aorta blood flow by magnetic resonance imaging during supine cycling exercise of increasing intensity

**DOI:** 10.1186/1532-429X-15-S1-P239

**Published:** 2013-01-30

**Authors:** Laura Ellwein, John F LaDisa, Stacy Leibham, Sheila Schindler-Ivens, Margaret M Samyn

**Affiliations:** 1 Mathematics and Applied Mathematics, Virginia Commonwealth University, Richmond, VA, USA; 2 Biomedical Engineering, Marquette University, Milwaukee, WI, USA; 3 Pediatrics, Herma Heart Center, Children's Hospital of Wisconsin, Milwaukee, WI, USA; 4 Radiology, Children's Hospital of Wisconsin, Milwaukee, WI, USA; 5 Physical Therapy, Marquette University, Milwaukee, WI, USA; 6 Medicine, Division of Cardiovascular Medicine, Medical College of Wisconsin, Milwaukee, WI, USA

## Background

Cardiac magnetic resonance imaging (MRI) may be used for the diagnosis and follow-up of diseases affecting the thoracic aorta (TA). Typically, patients are imaged at rest. Quantifying TA blood flow distribution during activity may improve understanding of disease impact by assessing possible factors contributing to exercise intolerance. Blood flow quantification during exercise MRI, though, has heretofore been complicated by prolonged MRI scan times, motion artifact, and gradient field inhomogeneities. We developed a protocol for phase-contrast (PC) MRI flow quantification in the TA and head and neck arteries during three-tiered supine cycling submaximal exercise.

## Methods

PC-MRI was acquired in the ascending aorta (AAo) and innominate (IA), left common carotid (LCCA), and left subclavian (LSA) arteries using a 1.5T Siemens MAGNETOM® Symphony magnet. Six healthy volunteers (28±2 years) were imaged during rest and supine pedaling of an MRI-compatible cycle to reach 130%, 150%, and 170% of resting heart rate (HR_130_, HR_150_, HR_170_). Scan parameters balanced image quality with acquisition time, accounting for subject positioning in the magnet and motion from pedaling. Time-resolved volumetric blood flow was calculated from PC-MRI data and estimated in the descending aorta (dAo). Flow quantification included cardiac index from AAo flow, arterial normalized mean flow (NMF,L/min/m^2^) and flow distribution (FD,% of AAo flow). Significance of flow quantification between HRs and rest was tested post-hoc with Student's t-test.

## Results

Cardiac index increased linearly (r^2^=0.99) from 3.1±0.3 to 5.2±0.3 L/min/m^2^ from rest to HR_170_ (p<0.05). HR increased to 111±5.9 bpm and workload to 38±9 watts at HR_170_. In the dAo, NMF increased linearly with respect to HR (r^2^=0.99) by 46%, 72%, and 93% with significance at all levels (Table 1). The IA showed significant increase in NMF of 39% at HR_170_ compared to rest. Increases in NMF in the LCCA and LSA were not significant. FD in the dAo increased significantly by 12% and 11% at HR_150_ and HR_170_ compared to rest, with concomitant decreases in the LCCA (27%, 31%) and LSA (25%, 20%). FD in the IA decreased at each HR level but without significance.

**Table 1:** Flow quantification (mean ± SE, n=6) in the descending aorta (dAo), innominate (IA), left common carotid (LCCA), and left subclavian (LSA) arteries. (Statistically significant results are in bold, p<0.05)

	NMF, L/min/m^2^				FD, % of AAo flow			
	Rest	HR_130_	HR_150_	HR_170_	Rest	HR_130_	HR_150_	HR_170_

dAo	2.12±0.23	**3.00±0.24**	**3.51±0.22**	**3.91±0.23**	67±1.6%	72±1.5%	**76±0.1%**	**75±1.3%**

IA	0.50±0.03	0.59±0.07	0.59±0.07	**0.69±0.07**	16±0.8%	14±1.0%	13±1.0%	13±1.0%

LCCA	0.23±0.01	0.27±0.02	0.24±0.02	0.26±0.03	7.6±0.7%	6.5±0.4%	**5.3±0.6%**	**5.0±0.5%**

LSA	0.28±0.04	0.34±0.05	0.30±0.04	0.38±0.06	8.7±0.9%	7.9±0.7%	**6.4±0.6%**	**7.0±2.0%**

NMF: Normalized mean flow; FD: Flow distribution.

## Conclusions

This investigation marks the first known flow quantification in the TA and head and neck arteries during supine exercise cardiac MRI. These pilot data show the changes in cardiac output and preferential redistribution of blood flow during increasing exercise intensity in a group of healthy young adults. Future studies using the protocol introduced here can now be conducted for various populations with TA disease.

## Funding

This work was supported by an AHA Postdoctoral Fellowship award 10POST4210030 (to LME) and an NIH grant R15HL096096-01 (to JFL).

